# Supplementary figures and images for: Hsa_circ_0006834 represses intrahepatic cholangiocarcinoma proliferation through activating AMPK-mTOR pathway and autophagy via has-miR-637-NGFR network
Source: PLoS One. 2025 Aug 4;20(8):e0329847. doi: 10.1371/journal.pone.0329847 (PMC12321115; doi:10.1371/journal.pone.0329847)

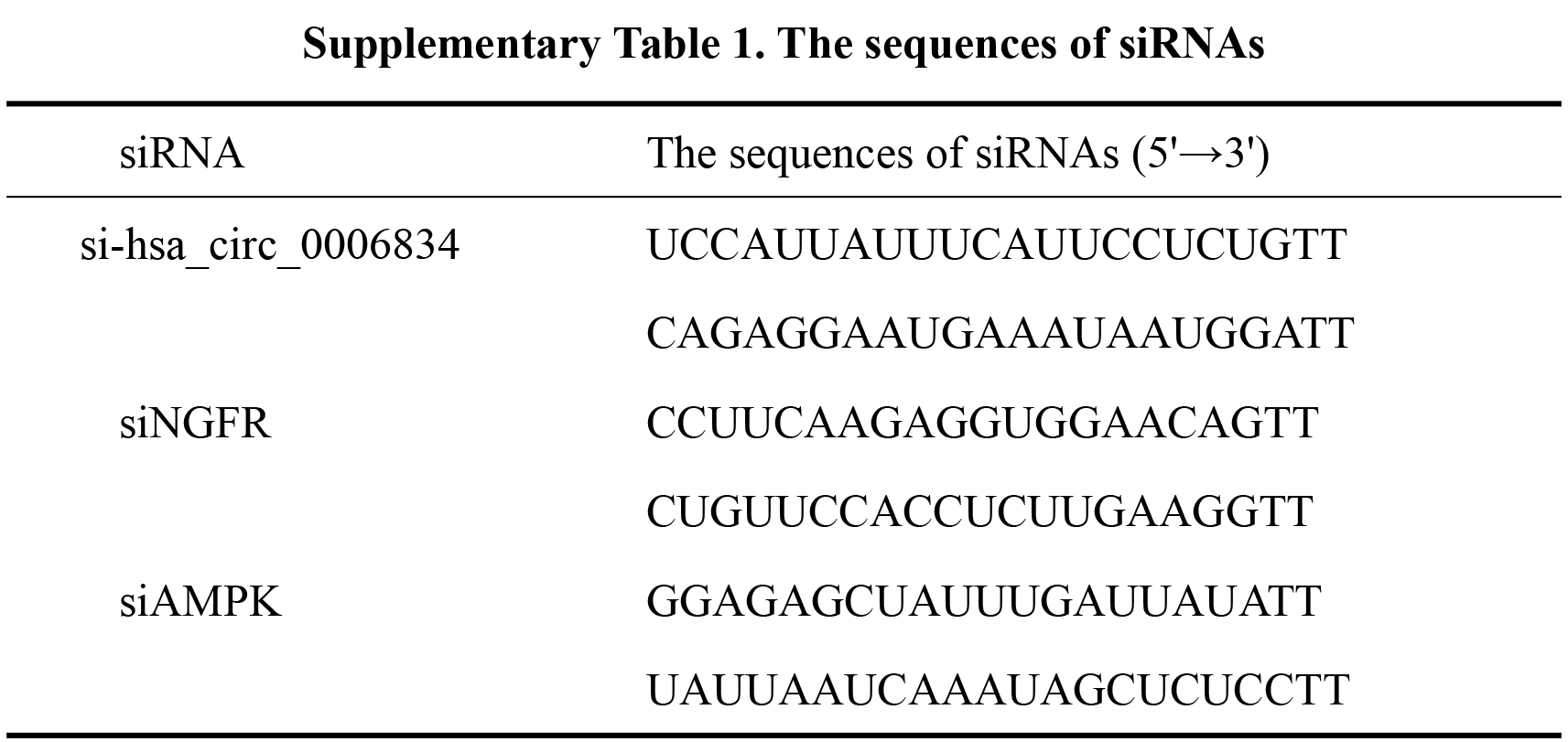

Supplement: S1 Table — (JPG) [file pone.0329847.s001.jpg]

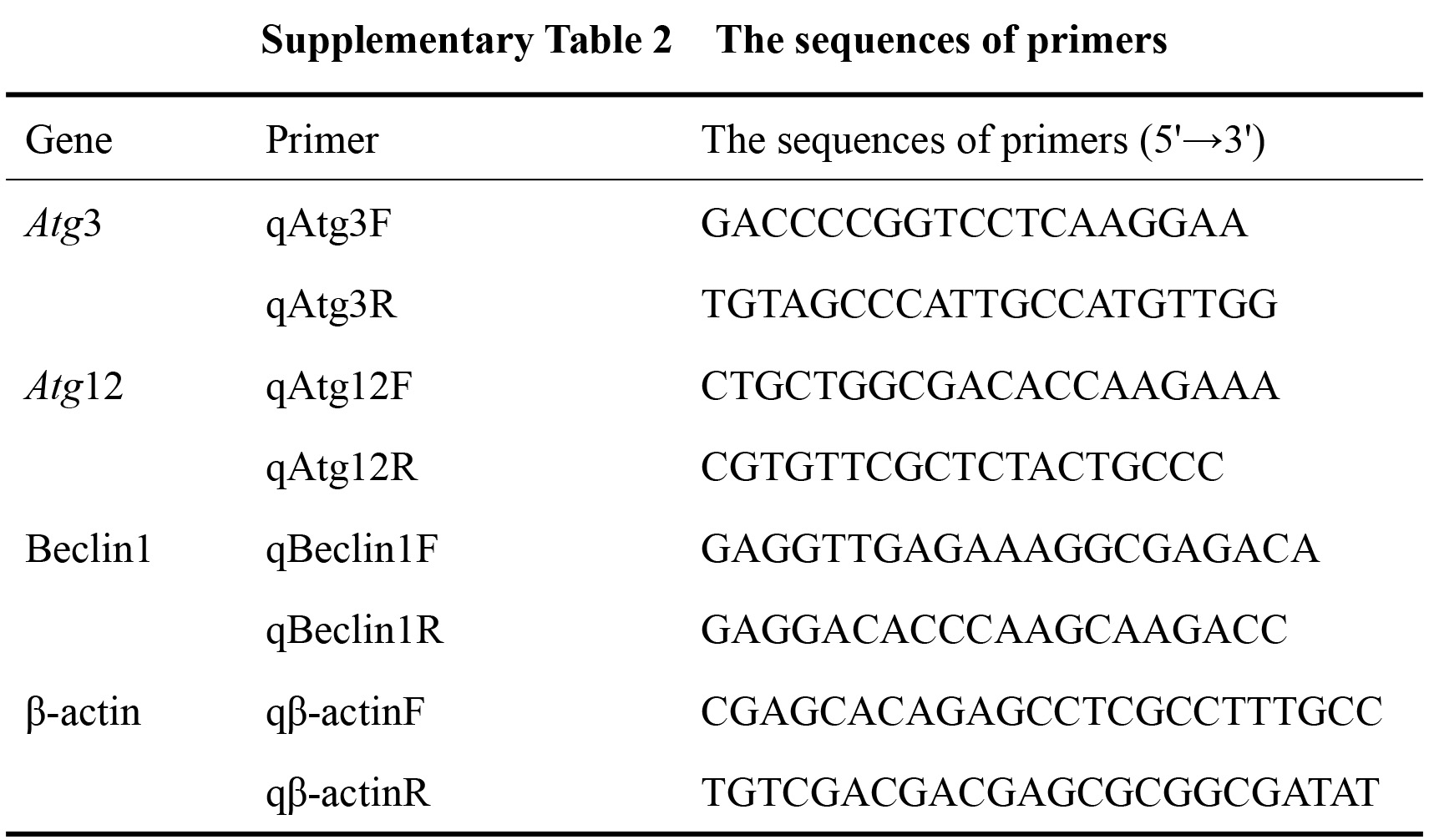

Supplement: S2 Table — (JPG) [file pone.0329847.s002.jpg]

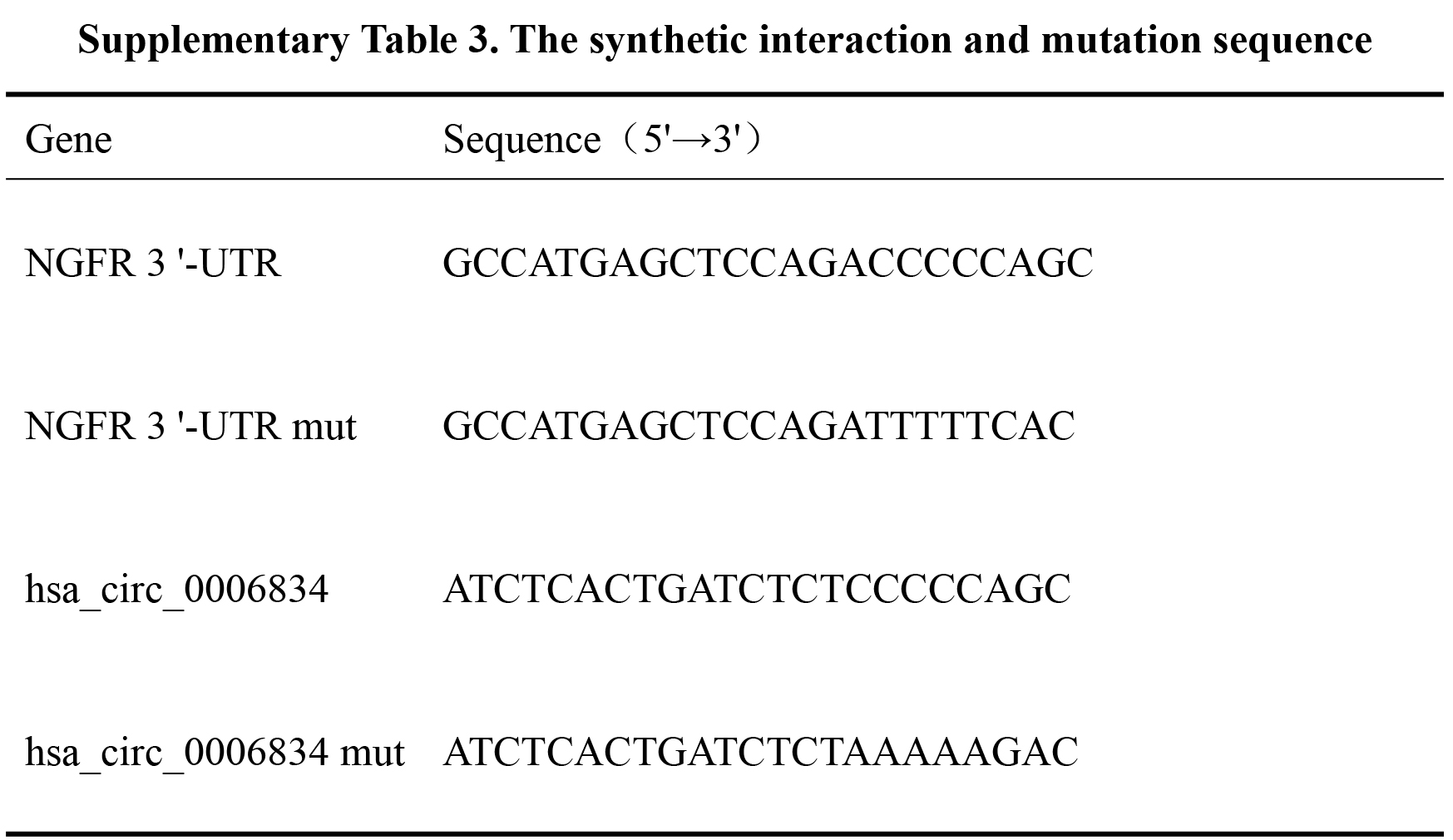

Supplement: S3 Table — (JPG) [file pone.0329847.s003.jpg]
