# Supplementary material for: Hsa_circ_0006834 represses intrahepatic cholangiocarcinoma proliferation through activating AMPK-mTOR pathway and autophagy via has-miR-637-NGFR network
Source: PLoS One. 2025 Aug 4;20(8):e0329847. doi: 10.1371/journal.pone.0329847 (PMC12321115; doi:10.1371/journal.pone.0329847)

Original images for Western blot in Fig. 2C

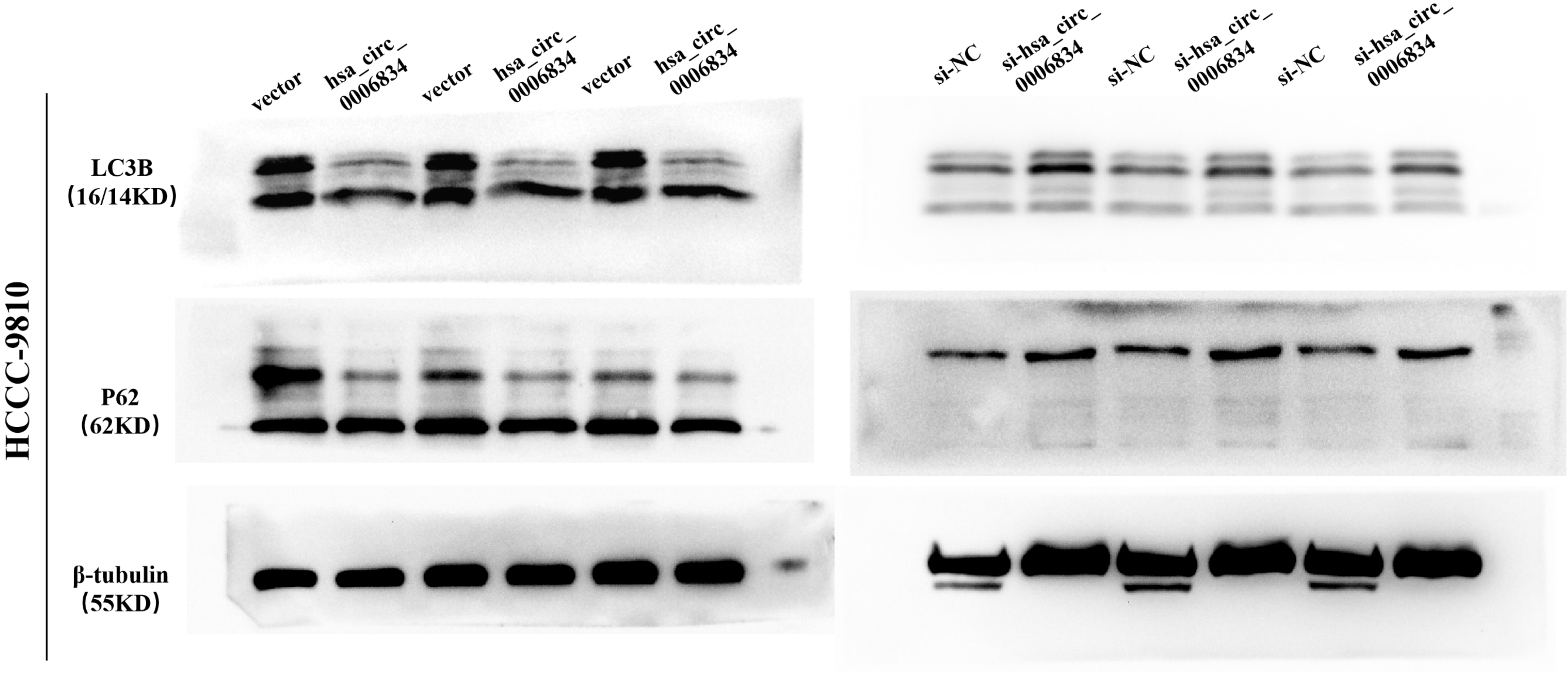

Original images for Western blot in Fig. 2D

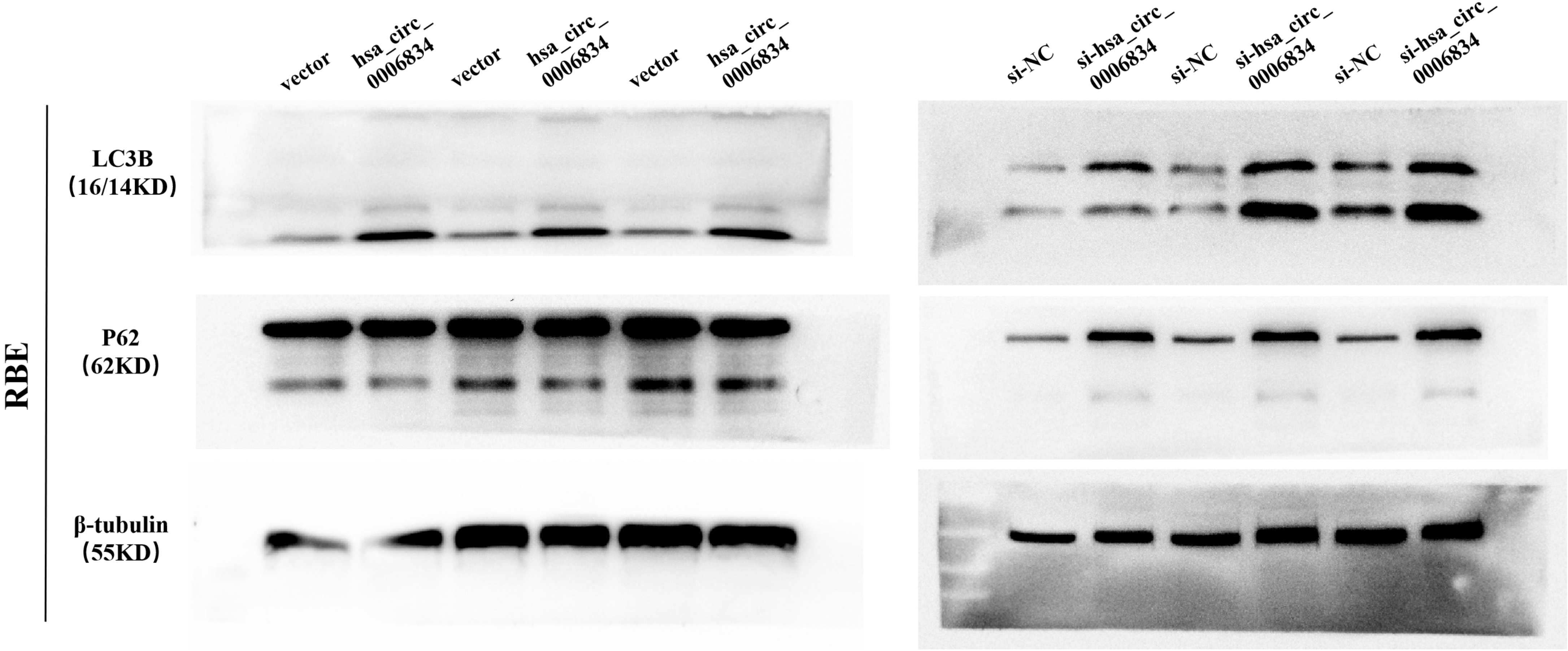

Original images for Western blot in Fig. 3K

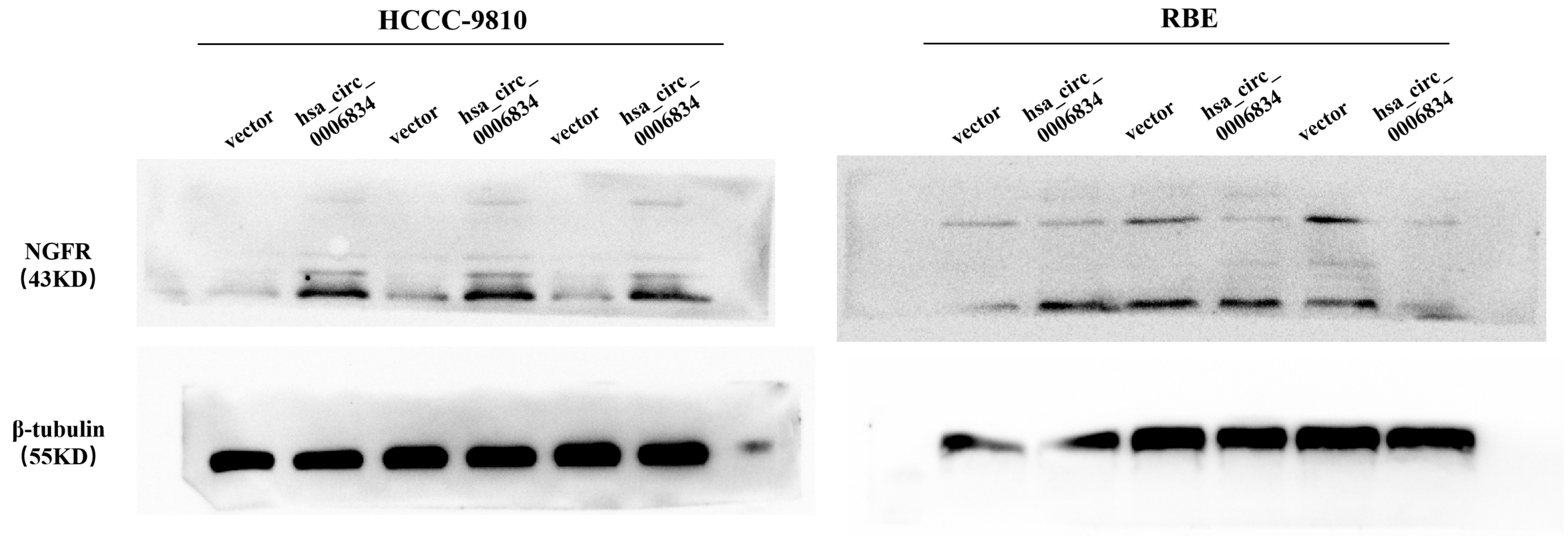

Original images for Western blot in Fig. 4C

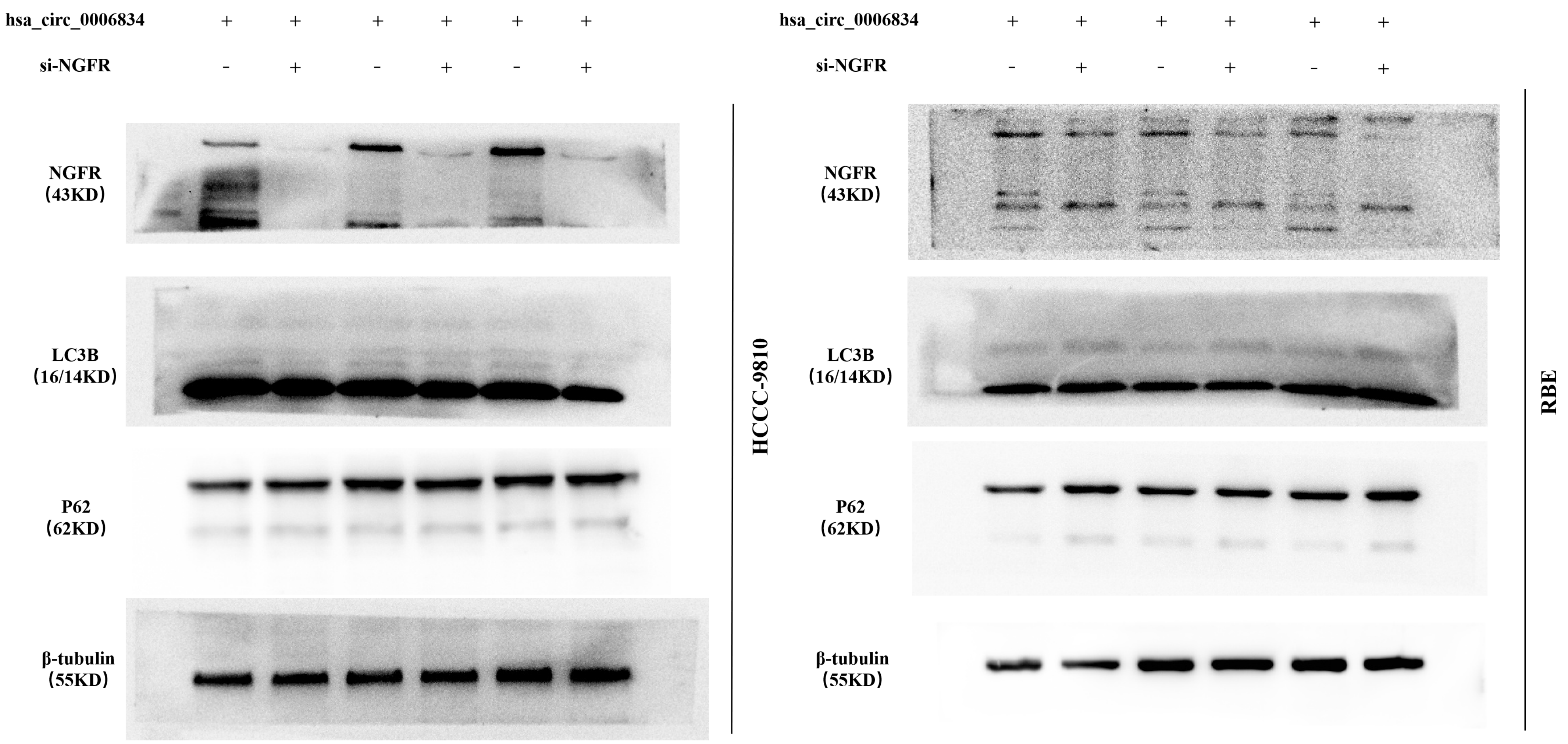

Original images for Western blot in Fig. 5A

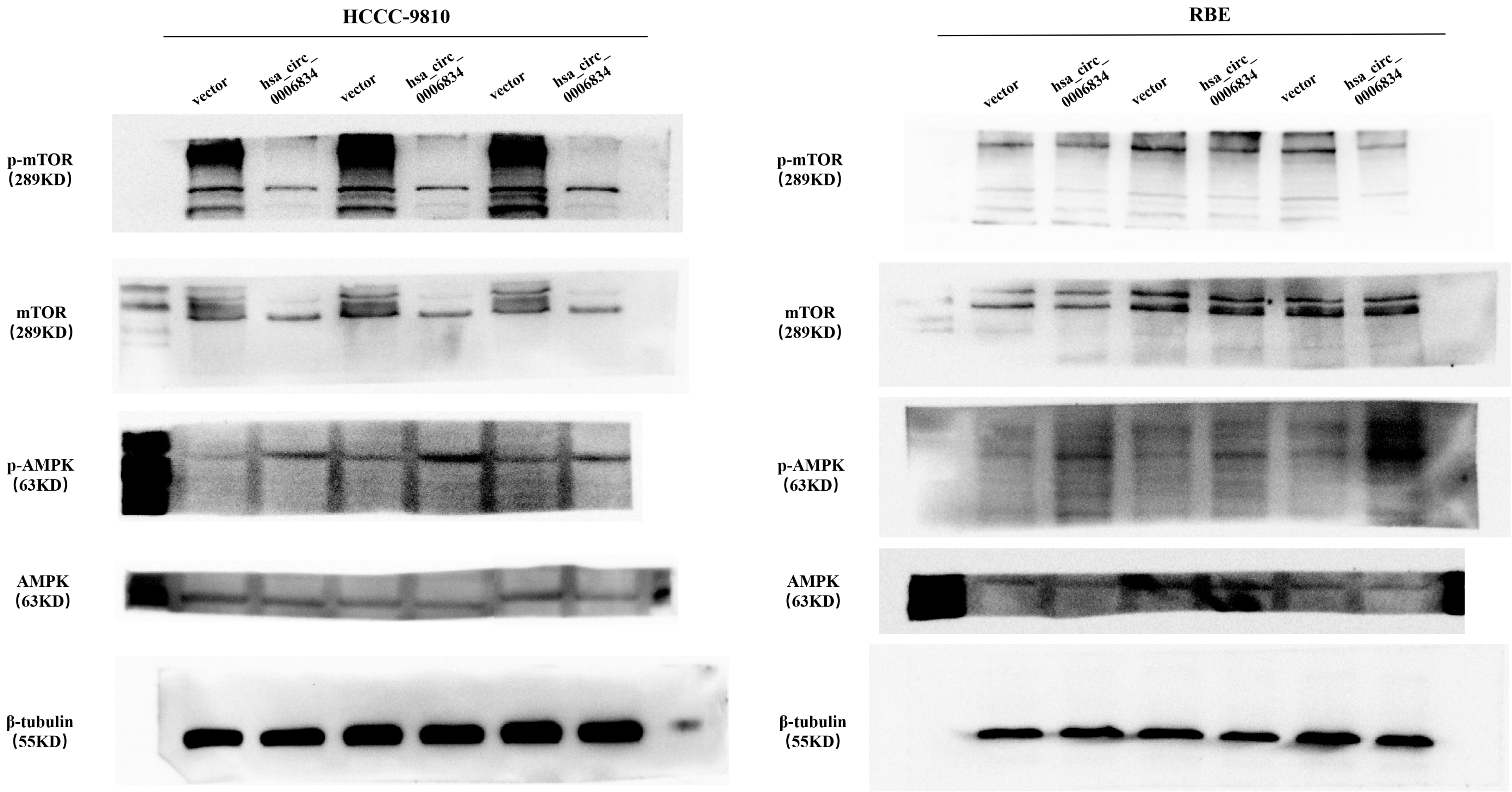

Original images for Western blot in Fig. 5C

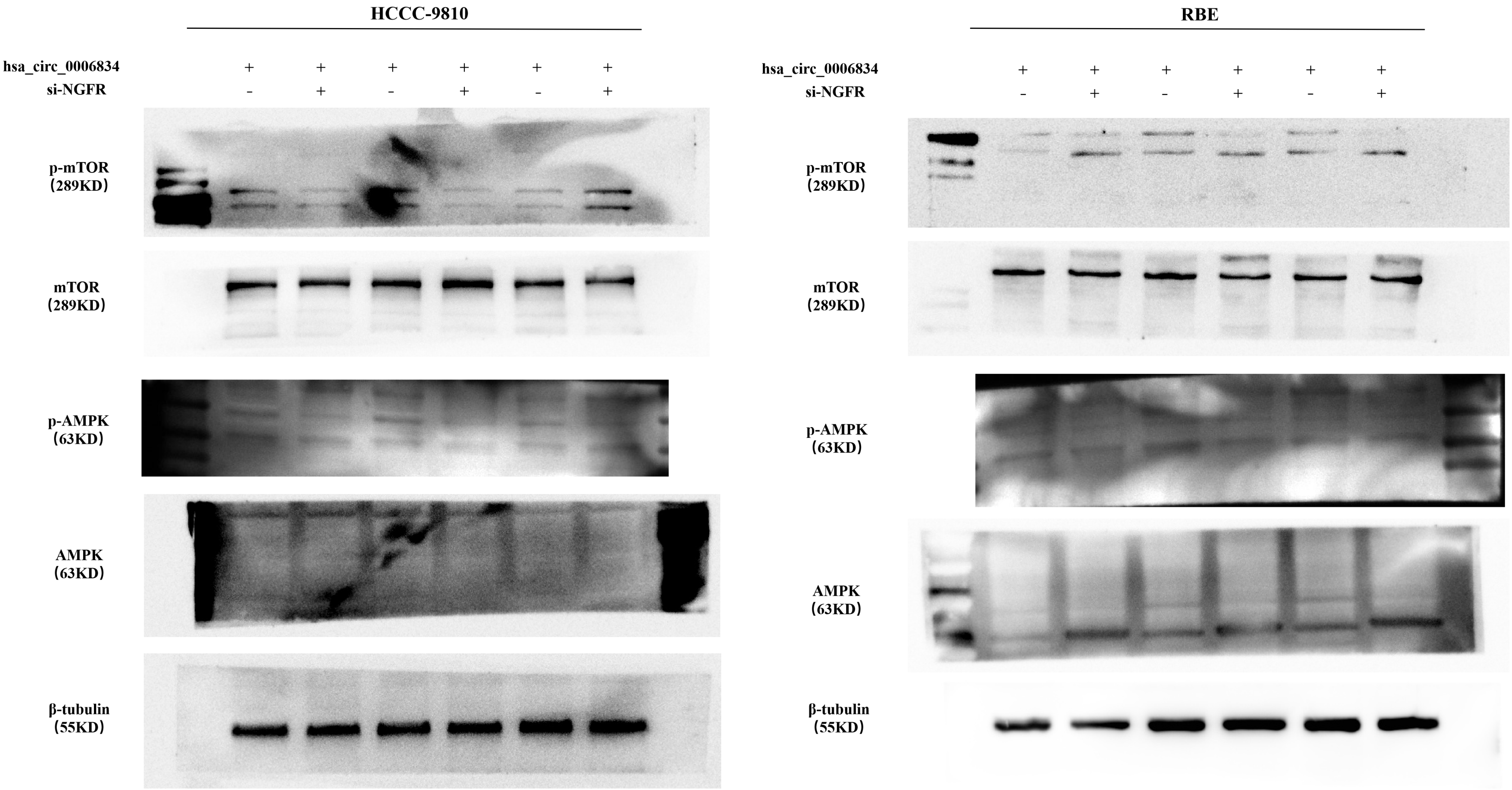

Original images for Western blot in Fig. 6A

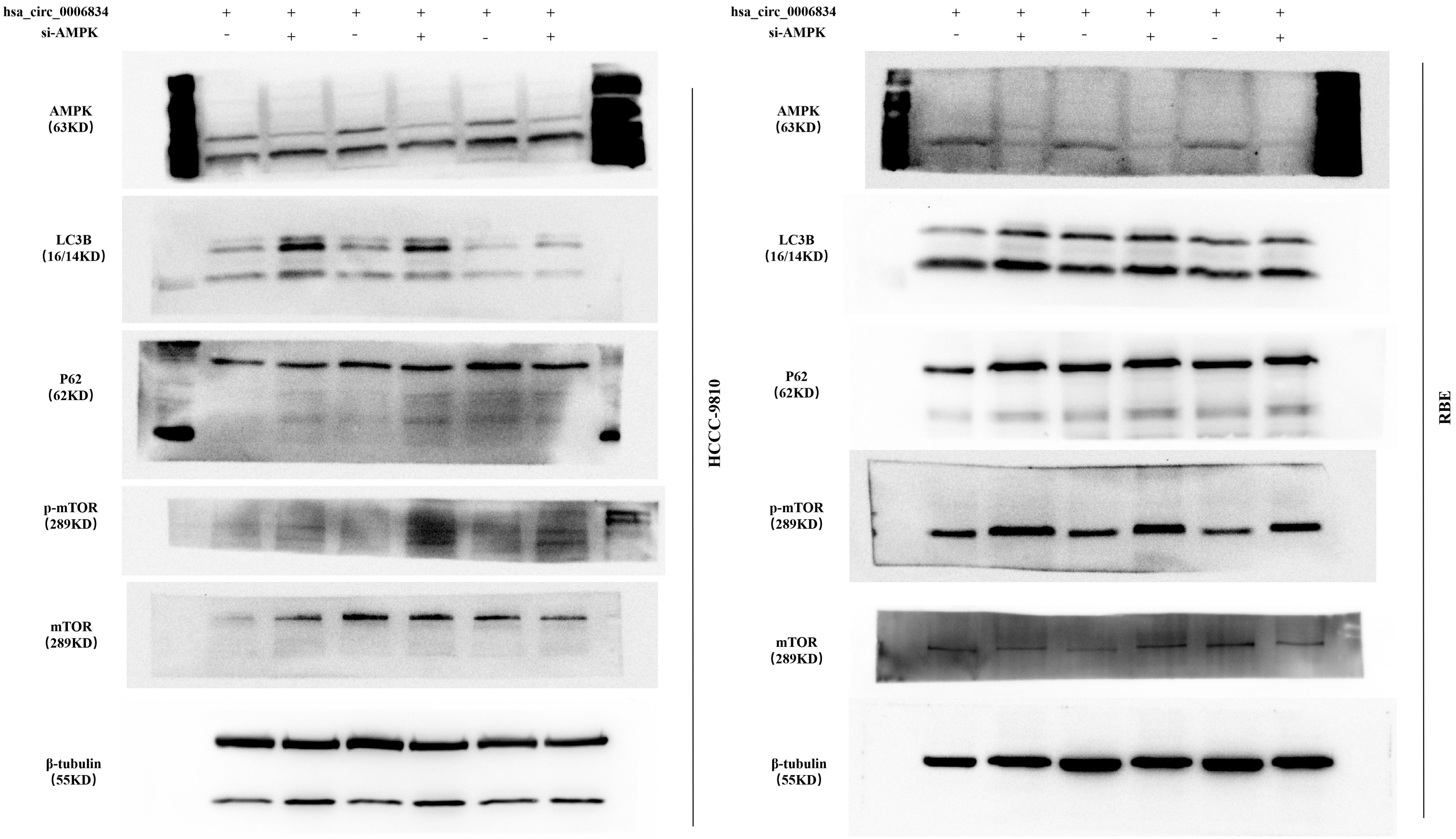

Supplement: S4 File — (PDF) [file pone.0329847.s004.pdf]
