# Supplementary material for: Hsa_circ_0006834 represses intrahepatic cholangiocarcinoma proliferation through activating AMPK-mTOR pathway and autophagy via has-miR-637-NGFR network
Source: PLoS One. 2025 Aug 4;20(8):e0329847. doi: 10.1371/journal.pone.0329847 (PMC12321115; doi:10.1371/journal.pone.0329847)
Supplement: Attachment — Submitted filename: PONE-D-25-12011_review.docx [file pone.0329847.s005.docx]

This manuscript investigates the role of a circular RNA, hsa_circ_0006834, in intrahepatic cholangiocarcinoma (iCCA). The study combines in vitro experiments using iCCA cell lines to propose a mechanism where hsa_circ_0006834 functions as a competing endogenous RNA (ceRNA). The authors suggest that hsa_circ_0006834 sponges has-miR-637, leading to the upregulation of its target, nerve growth factor receptor (NGFR). This, in turn, is proposed to activate the AMPK-mTOR signaling pathway, induce autophagy, and ultimately suppress iCCA cell proliferation, migration, and invasion. While the manuscript presents a cohesive and interesting narrative with a logical series of experiments, several significant aspects require further experimental validation and clarification to strengthen the conclusions. The manuscript also needs improvements in its structure, logical flow, and linguistic precision.

**Major revisions are required:**

- To more robustly establish the proposed ceRNA mechanism, have the authors considered performing critical rescue experiments? For instance, could the inhibitory effects of hsa_circ_0006834 overexpression on cell proliferation, migration, and autophagy be reversed by the co-transfection of a has-miR-637 mimic? This would provide stronger evidence that the circRNA's function is mediated primarily through sponging this specific miRNA.
- The RNA pull-down assay demonstrated an enrichment of miR-637 with the biotinylated hsa_circ_0006834 probe. To confirm the specificity of this interaction, it would be beneficial to include a control using a mutated biotinylated probe that disrupts the predicted miR-637 binding site. This would demonstrate that the interaction is sequence-specific.
- While the data show that silencing NGFR reverses the effects of hsa_circ_0006834 overexpression, the mechanism linking NGFR to AMPK activation is not directly elucidated. The manuscript states NGFR is associated with the pathway, citing another study. Can the authors provide more direct mechanistic insight in their specific iCCA cell model? For example, does NGFR interact with AMPK or an upstream kinase?
- The study convincingly shows that silencing AMPK reverses the pro-autophagic and anti-proliferative effects of hsa_circ_0006834. However, in these AMPK knockdown experiments, has the phosphorylation status of downstream mTOR targets, such as p70S6K or 4E-BP1, been assessed to further confirm the modulation of the entire AMPK-mTOR axis?
- The manuscript relies heavily on two iCCA cell lines (HCCC-9810 and RBE). While this is a standard approach, have the authors validated their key findings, such as the expression levels of hsa_circ_0006834, miR-637, and NGFR, in a cohort of human iCCA patient tissues versus adjacent non-tumorous tissues? This would significantly enhance the clinical relevance of the findings. The manuscript mentions that NGFR was lower in tumor tissues but the data presentation is limited.
- The introduction is adequate but could be more focused. It broadly discusses autophagy, AMPK-mTOR, and circRNAs. It would be more impactful to streamline this section to build a stronger, more direct rationale for investigating the specific hsa_circ_0006834/miR-637/NGFR axis in the context of iCCA from the outset.
- The references cited in this article are not sufficient, and there is a lack of in-depth comparative discussion. Background and methodology also require further literature support. Some related research should be cited:

10.15212/bioi-2022-0010

10.15212/bioi-2024-0013

10.15212/bioi-2024-0012

10.15212/bioi-2024-0066

10.34133/research.0080

10.34133/research.0033

10.5847/wjem.j.1920-8642.2022.015

10.1016/j.celrep.2024.114697

10.53388/tmr20220207263

10.53388/tmr20211227255

- There are several typographical errors and inconsistencies within the figure legends that detract from the manuscript's quality. For instance, the legend for Figure 1 refers to "Fig. 1F-G" while the figure itself only has panels up to F. Similarly, the legend for Figure 2 refers back to statistical graphs in "Fig. 1E-F", and Figure 3's legend refers to "Fig. 2L". The entire manuscript requires careful proofreading for such errors.
- The terminology for key reagents is inconsistent. For example, the circRNA is referred to as "hsa_circ_0006834", "circRNA_0006834", and "hsa circ 0006834" throughout the text and figures. Please select a single, consistent nomenclature and apply it throughout the manuscript for clarity.
- The Discussion section effectively summarizes the results but would be strengthened by a more critical analysis of the findings. Please include a subsection discussing the limitations of the study, such as the reliance on in-vitro data and the possibility that this circRNA may have other functions or interact with other molecules beyond the specific axis investigated. This would provide a more balanced perspective.
- The conclusion that hsa_circ_0006834 represses iCCA proliferation through activating the AMPK-mTOR pathway is a strong claim. While the data link these events, causality is implied but not definitively proven to be the sole mechanism. The authors should consider moderating this language to state that hsa_circ_0006834 is associated with or contributes to the activation of this pathway, which in turn suppresses proliferation. This would more accurately reflect the nature of the evidence provided.
